# Supplementary material for: The interactome of intact mitochondria by cross-linking mass spectrometry provides evidence for coexisting respiratory supercomplexes
Source: Mol Cell Proteomics. 2017 Dec 8;17(2):216–32. doi: 10.1074/mcp.RA117.000470 (PMC5795388; doi:10.1074/mcp.RA117.000470)
Supplement: Supplemental Data [file supp_RA117.000470_133922_0_supp_23978_fzffwf.zip › spectra_annotation/mito_DR_spectra_annotation/149-1-33-1-1-1.pdf]

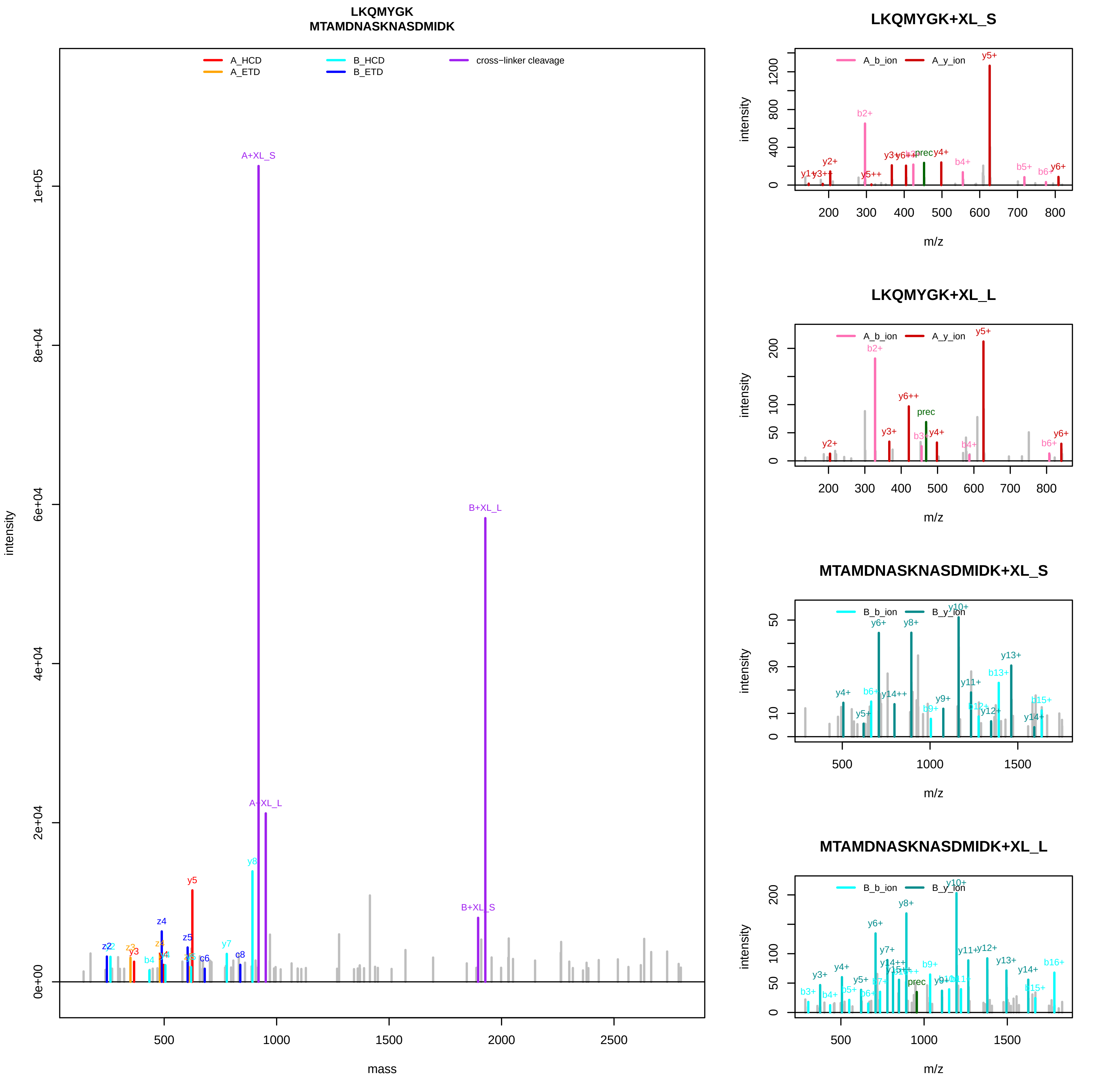

LKQMYGK
MTAMDNASKNASDMIDK
A_HCD
A_ETD
B_HCD
B_ETD
cross−linker cleavage
A+XL_S
B+XL_L
A+XL_L
B+XL_S
y8
y5
y7
z4
z5
c6
c8
z2
z3
y3
b4
intensity
mass
0e+00
2e+04
4e+04
6e+04
8e+04
1e+05
500
1000
1500
2000
2500
LKQMYGK+XL_S
A_b_ion
A_y_ion
y5+
b2+
y2+
y4+
prec
b4+
b5+
b6+
y6+
y5++
intensity
m/z
LKQMYGK+XL_L
A_b_ion
A_y_ion
y5+
b2+
y6++
prec
y3+
y4+
y2+
b4+
b6+
y6+
intensity
m/z
MTAMDNASKNASDMIDK+XL_S
B_b_ion
B_y_ion
y10+
y6+
y8+
y13+
b13+
y11+
y4+
y14++
y9+
b9+
y12+
b15+
y14+
y5+
intensity
m/z
MTAMDNASKNASDMIDK+XL_L
B_b_ion
B_y_ion
y10+
y8+
y6+
y7+
y11+
y12+
y13+
b9+
b16+
y14+
y4+
y3+
y5+
b15+
b3+
b4+
b5+
b6+
prec
intensity
m/z
